# Green space exposure and risk of amyotrophic lateral sclerosis: a population-based case-control study in Northern Italy

**DOI:** 10.1186/s12940-026-01311-w

**Published:** 2026-05-22

**Authors:** Asia Sarti, Jessica Mandrioli, Sofia Costanzini, Guigo Xavier Balbo, Carlotta Malagoli, Niccolò Martini, Francesca Despini, Federica Violi, Marcella Malavolti, Ilaria Martinelli, Matteo Giacchino, Giulia Donelli, Elena Canali, Lucia Zinno, Sergio Teggi, Marco Vinceti, Tommaso Filippini

**Affiliations:** 1 Environmental, Genetic and Nutritional Epidemiology Research Center (CREAGEN), Department of Biomedical, Metabolic and Neural Sciences, University of Modena and Reggio Emilia, Modena, 41125 Italy; 2 Department of Biomedical, Metabolic and Neural Sciences, University of Modena and Reggio Emilia, Via Campi, 287, Modena, 41125 Italy; 3 Department of Neurosciences, Azienda Ospedaliero Universitaria di Modena, Modena, 41126 Italy; 4 Department of Engineering “Enzo Ferrari”, University of Modena and Reggio Emilia, Modena, 41125 Italy; 5 Local Health Authority, Reggio Emilia and Arcispedale S. Maria Nuova, IRCCS, Reggio Emilia, Italy; 6 Department of Neurology, IRCCS Arcispedale Santa Maria Nuova, Reggio Emilia, 42123 Italy; 7 Department of General and Specialized Medicine, University Hospital of Parma, Parma, 43126 Italy; 8 Department of Epidemiology, Boston University School of Public Health, Boston, MA 02118 USA; 9 School of Public Health, University of California Berkeley, Berkeley, CA 94074 USA

**Keywords:** Amyotrophic lateral sclerosis, Environmental factors, Greenness, Satellite-based indices, Land use indices

## Abstract

**Background:**

The contribution of environmental determinants in the etiology of amyotrophic lateral sclerosis (ALS) is still unclear. Among the various environmental factors, exposure to green spaces, also known as greenness, is attracting considerable interest as many studies have reported its beneficial associations to health outcomes, particularly to neurodegenerative diseases.

**Methods:**

To investigate the relation between greenness and ALS risk, we conducted a population-based case-control study in a Northern Italy population (from Modena, Reggio Emilia and Parma provinces), including 499 cases of ALS newly-diagnosed from 1998 to 2011 and 1,935 sex-, age-, and province-matched controls randomly selected from study provinces residents. We evaluated the association between greenness in the proximity of residence and ALS risk, assessing exposure through multiple satellite-based and land-use derived indices, both conventional and novel devised, for a total of six indices, each providing specific information, including annual and seasonal Normalized Difference Vegetation Index (NDVI), NDVI-weighted to green areas, green cover ratio, accessibility index, and their combined Green Exposure Index (GEI). We used conditional logistic regression models to evaluate disease risk for increasing exposure through both fixed-categories and non-linear restricted cubic splines.

**Results:**

We observed a non-linear U-shaped association between greenness and ALS risk with increased odds ratios at both low and high levels. Results were more defined when using NDVI-based indices, while the associations were smoother when considering GEI. The higher risk at low levels may be related to lower accessibility to green spaces with lower physical activity and higher exposure to outdoor air pollutants, whilst elevated greenness may reflect higher exposure to neurotoxic pesticides. These results were confirmed also after adjustment for potential confounders, namely magnetic fields and light at night. Sex stratified analysis yielded similar results, except for more distinct associations in females for GEI.

**Conclusions:**

Despite the limitations due to possible unmeasured confounding and exposure misclassification related to the use of residential data, our results provide evidence of an inverse association between intermediate residential greenness and ALS risk, and may have public health implications including disease prevention and urban planning.

**Supplementary Information:**

The online version contains supplementary material available at 10.1186/s12940-026-01311-w.

## Background

Amyotrophic lateral sclerosis (ALS) is a fatal neurodegenerative disorder affecting upper and lower motor neurons. The motor neurons degeneration leads to progressive limb muscles weakness, bulbar dysfunction with swallowing impairment and respiratory failure, and ultimately paralysis [1, 2]. The most common form of ALS is the sporadic one, accounting for approximately 85–90% of cases, while only about 10–15% have familial history [3, 4]. ALS has an incidence of approximately 2–3 cases per 100,000 person-year, shows marked heterogeneity worldwide, and is associated with a median survival ranging from 2 to 5 years from symptoms onset [5, 6].

The etiology of ALS remains largely unknown and is thought to involve a complex interplay between genetic susceptibility and environmental exposures [7, 8]. While the role of genetic factors is relatively well established, the contribution of environmental determinants remains less clearly defined, despite extensive investigation [9–12]. Environmental exposures that have been implicated include intense physical activity, trauma, exposure to pesticides, heavy metals and selenium, electromagnetic fields, cyanotoxins, and air pollution, among others [13–23].

Exposure to green spaces (or ‘greenness’) is among those environmental factors currently of considerable interest in epidemiology with reference to neurodegenerative diseases. Various techniques and indices can be used to evaluate and represent greenness exposure. The approaches most commonly used to quantify greenness are based on satellite images, as in the case of the Normalized Difference Vegetation Index (NDVI) metric, and land cover datasets [24]. NDVI-based metrics mainly capture vegetation biomass and density, whereas approaches based on land use or land cover maps enable the identification and classification of different types of green spaces according to land use categories. It has been demonstrated that residential greenness provides beneficial effects on human health [25]. Green spaces may exert beneficial effects through several mechanisms, including psychological rest, stress reduction, promotion of physical activity, enhancement of social interaction and mitigation of environmental risk factors, such as air pollution [26–28]. Decreased mortality for neurodegenerative diseases has been reported at increasing exposure to residential greenness [29, 30]. Similarly, lower incidence of Parkinson’s disease [31] and its hospitalization have been reported for increased exposure to green spaces [32]. Thereby, an inverse relation also with ALS risk could be expected. However, no study has so far specifically investigated the risk of ALS. In this study, we aimed to evaluate the relation between greenness exposure, measured through several different indices, and ALS risk in an Italian population.

## Methods

### Study population

We carried out a case-control study encompassing three provinces of the Emilia-Romagna region in Northern Italy (Modena, Parma and Reggio-Emilia), with a total of approximately 1,700,000 inhabitants in 2000. This study was approved by the Modena Hospital Ethics Committee (approval no. 80.11 on May 26, 2011). We identified all the patients receiving an ALS diagnosis from 1998 to 2011 through administrative data sources such as the ALS Emilia-Romagna Registry [33], the Emilia-Romagna region hospital discharge directory [34], drug prescription directory and death certificate directory. Only ALS cases with a “definite” or “probable” diagnosis, as defined by El Escorial revised criteria, were included in the study [35]. We randomly selected sex-, age-, and province of residence-matched controls (with a variable criterion of 1:3 or 1:4) from the database of residents of the respective provinces managed by the Local Health Authorities, as described in detail elsewhere [36, 37].

### Geocoding of study participants

The residential address of each case at date of diagnosis and of their matched controls, at the corresponding year, were obtained from the database of the Ministry of Finance Revenue Agency, and then residential history was rebuilt, taking as eligible residences those maintained for at least two years. We geocoded participants’ residences using *OpenStreetMap* database, and *Google Earth Pro* when geographic coordinates could not be found through the former source. When geographic coordinates were still not accessible, we directly geocoded them in loco by using a portable GPS device (Garmin GPSmap 60CSx, Garmin Int. Corp., Olathe, KS). We finally inserted the georeferenced data into a GIS (Geographic Information System) platform, using the QGIS software (QGIS Development Team, 2024) [38]. To obtain maps of the environmental exposure to greenness, we used a methodology we recently developed [39].

### Greenness assessment

Greenness exposure in the proximity of residence was assessed using different indices previously developed [39], each enabling to access a specific dimension of the green spaces characteristics: Green Coverage Ratio (GCR), Accessibility (A_i_), Normalized Difference Vegetation Index (NDVI) and Green Exposure Index (GEI). The land use based GCR quantifies the amount of vegetation within an area of interest providing vegetation quantity information [40]. A_i_ allows to estimate availability to green spaces, based on the 15 min walkability criterion, as a function of both distance and accessibility of green areas obtained through land use maps [41]. NDVI is a satellite-based index that provides quantitative measures of vegetation density and quality, ranging from − 1 to + 1, with negative and positive values indicating presence of water bodies and vegetation, respectively [42]. Values below 0.1 correspond to sparsely or non-vegetated areas (e.g., barren areas of rock, sand, or snow), intermediate values (0.2–0.5) to moderately vegetated areas (e.g., shrubs, grasslands, parks and off-season crops), and above 0.6 to healthy and dense vegetation (e.g., forests or crops at their peak of growth) [43]. We exploited additional NDVI versions, specifically a Spring seasonal one (i.e., considering April, May and June only: NDVI_amj_), and a version accounting only for green areas, according to land use characterization within the buffer (NDVI_green_). Finally, we also used a newly proposed index that considers this multidimensionality of greenness, combining the information on vegetation mentioned above and hopefully providing a more comprehensive evaluation of the exposure, GEI [39]. This model involves the sum of two weighted terms, one related to vegetation and one to accessibility, obtained respectively upon the indices mentioned above (specifically GCR, NDVI_green_, having been identified as the most suitable vegetation proxy among the ones tested in this study, and A_i_). We used three scenarios for GEI, respectively GEI_1_, GEI_2_ and GEI_3_, with different weighting factors applied to the vegetation and accessibility terms, to probe its sensitivity and appreciate any underling trend.

The indices used and their main features are resumed in Table 1. A more detailed description of the indices characteristics and calculation procedure is provided in the Supplementary Material 1. To show visually their exposure pattern, we computed interpolation maps of the values of the investigated indices in the study areas (provinces of Parma, Reggio Emilia and Modena limits reported in Supplementary Figure S1) through a 50 m regular grid of virtual points. These maps were made using the Inverse Distance Weighting (IDW) method through the QGIS software (QGIS Development Team, 2024), exploiting the data acquired for year 2005. This year appeared to be the most representative one, having no data holes and being exactly in the middle of the time interval under study.

**Table 1:** Indices used for evaluation of exposure to greenness with a brief description of their main characteristics

Index	Features
GCR	Land cover-based index that quantifies the amount of vegetation within an area of interest.
A_i_	Accessibility index, based on the 15 min walkability criterion, that estimates availability of green spaces as a function of both distance and accessibility (determined on the base of land use type).
NDVI	Satellite-based index that expresses quantitative measures of vegetation density and health; this index is calculated using an annual time window and a 100 m radius circular buffer.
NDVI_amj_	Satellite-based index that expresses quantitative measures of vegetation density and health; this index is calculated using a Spring seasonal time window, specifically of April, May and June (amj) months, displaying the vegetation peak, and a 100 m radius circular buffer.
NDVI_green_	Satellite-based index that expresses quantitative measures of vegetation density and health; this index is calculated using a Spring seasonal time window, and a “green buffer” obtained through interpolation of 100 m radius circular buffers and land use maps.
GEI_1_	Comprehensive and multidimensional index integrating the above-mentioned ones (GCR, NDVI_green_, A_i_); first scenario highlighting the accessibility, with weights $\:{w}_{1}=0.3,\:{w}_{2}=0.7$.
GEI_2_	Comprehensive and multidimensional index integrating the above-mentioned ones (GCR, NDVI_green_, A_i_); second scenario empathizing the vegetation contribution with weights $\:{w}_{1}=0.7,\:{w}_{2}=0.3$.
GEI_3_	Comprehensive and multidimensional index integrating the above-mentioned ones (GCR, NDVI_green_, A_i_); third scenario with equal weight to both contributions ($\:{w}_{1}=0.5,\:{w}_{2}=0.5$).

### Data analysis

We estimated the risk to ALS in relation to greenness by calculating odds ratios (ORs) and their 95% confidence intervals (CIs) using a multivariate logistic regression model for matched data, i.e. sex, age and province of residence. We computed ALS risk through an exposure evaluation that exploits fixed categories of exposure divided in quartiles (< 25%; 25%-50%; 50%-75%; >75%), with the lowest quartile used as reference. We focused on the five years preceding the diagnosis. For each of the subjects we computed a mobile mean for the greenness indices, by considering the values at the year of diagnosis and at the previous five years from it. Additionally, we adjusted the multivariate logistic regression model for magnetic field, light at night (LAN) exposure, and for both, given the potential for confounding by these factors [37, 44]. We also modelled the non-linear relation among greenness and ALS through a restricted cubic spline regression model by selecting the optimal number of knots according to Akaike’s information criterion (AIC) and using the knot placement method recommended by Harrell [45]. Therefore, we used three knots at 10th, 50th and 90th percentiles, and the median value as reference in all analyses. To analyze data, we used the *clogit*, *mkspline* and *xblc* Stata-19.5 commands (Stata Corp., College Station, TX, USA, 2025).

## Results

Overall, we identified 499 ALS cases (235 in Modena, 152 in Reggio Emilia, 112 in Parma) and 1,935 matched controls. Mean age at disease onset was 67.2 years, specifically 65.1 years for males and 69.8 years for females. The distribution of the participants by sex, age at diagnosis and province of residence is shown in Table 2. Calculating the mean mobile, 10 subjects of the 2,434 showed a missing value for NDVI_green_, and consequently for GEI_1_, GEI_2_ and GEI_3_, being computed upon it. The median and interquartile range, stratified by category, of the greenness indices, considering the mean mobile associated to each subject, are reported in Table 2. The above-mentioned data stratified by sex are provided in Supplementary Table S1.

**Table 2:** Characteristics of study participants, stratified by category, for sex, age at diagnosis and province of residence; median and interquartile range (IQR) for cases and controls of GCR, A_i_, annual NDVI with 100 m radius buffer, spring seasonal NDVI_amj_ with 100 m radius buffer and spring seasonal NDVI_green_ with green buffer, GEI_1_ with weighting factors $\:{w}_{1}=0.3,\:{w}_{2}=0.7$, GEI_2_ with $\:{w}_{1}=0.7,\:{w}_{2}=0.3$and GEI_3_ with $\:{w}_{1}=0.5,\:{w}_{2}=0.5$

	Cases	Controls
	*N* (%)	*N* (%)
Total	499 (100.00)	1,935 (100.00)
Sex		
Male	269 (53.91)	1,038 (53.64)
Female	230 (46.09)	897 (46.36)
Age at diagnosis (years)		
Median (IQR)	68 (60–76)	68 (60–76)
< 55	74 (14.83)	281 (14.52)
55–74	273 (54.71)	1,063 (54.94)
≥ 74	152 (30.46)	591 (30.54)
Province		
Modena	235 (47.09)	905 (46.77)
Reggio Emilia	152 (30.46)	599 (30.96)
Parma	112 (22.44)	431 (22.27)
	**Median (IQR)**	**Median (IQR)**
GCR	0.102 (0–0.309)	0.098 (0–0.342)
A_i_	0.572 (0.460–0.730)	0.572 (0.460–0.730)
NDVI		
NDVI	0.397 (0.312–0.497)	0.391 (0.315–0.494)
NDVI_amj_	0.450 (0.362–0.575)	0.447 (0.361–0.571)
NDVI_green_	0.416 (0–0.592)	0.411 (0–0.575)
GEI		
GEI_1_	0.498 (0.400–0.582)	0.508 (0.400–0.583)
GEI_2_	0.351 (0.172–0.488)	0.350 (0.185–0.498)
GEI_3_	0.421 (0.286–0.542)	0.429 (0.286–0.536)

**Fig. 1:**
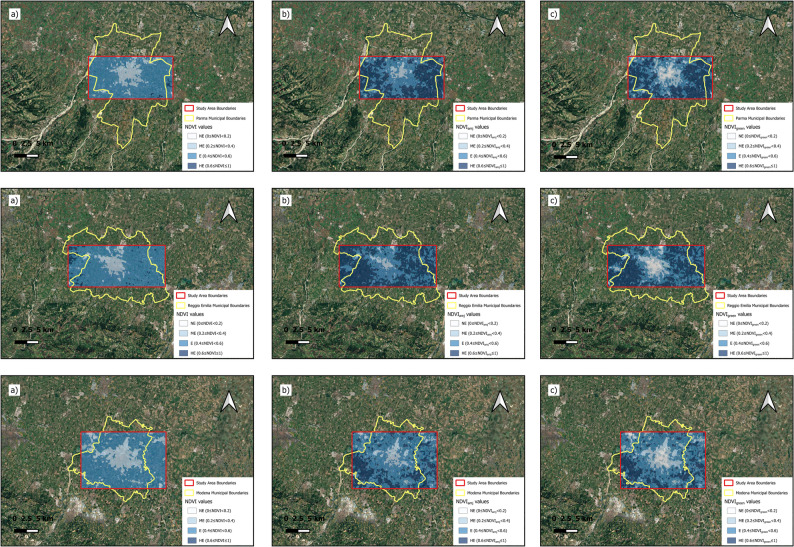
Map of interpolation in the study areas of Parma, Reggio Emilia and Modena of indices: (**a**) NDVI; (**b**) NDVI_amj_; (**c**) NDVI_green_

**Fig. 2:**
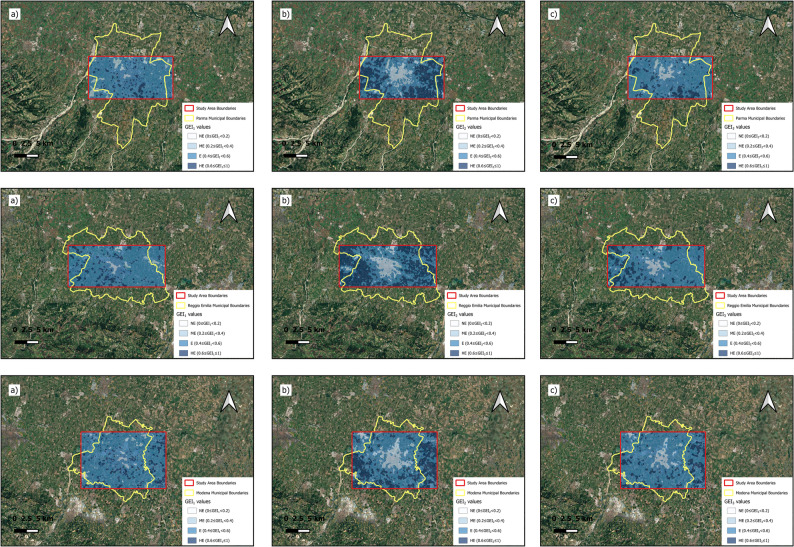
Map of interpolation in the study areas of Parma, Reggio Emilia and Modena of indices (**a**) GEI_1_; (**b**) GEI_2_; (**c**) GEI_3_

The interpolation maps of NDVI, NDVI_amj_, NDVI_green_, GEI_1_, GEI_2_ and GEI_3_ for Parma, Reggio Emilia and Modena are shown respectively in Figures 1 and 2 for NDVI and GEI. Lower exposure values, generally between 0.2 and 0.4 and even lower than 0.2 for NDVI_green_, characterized the urban areas, in accordance with vegetation distribution.

**Fig. 3:**
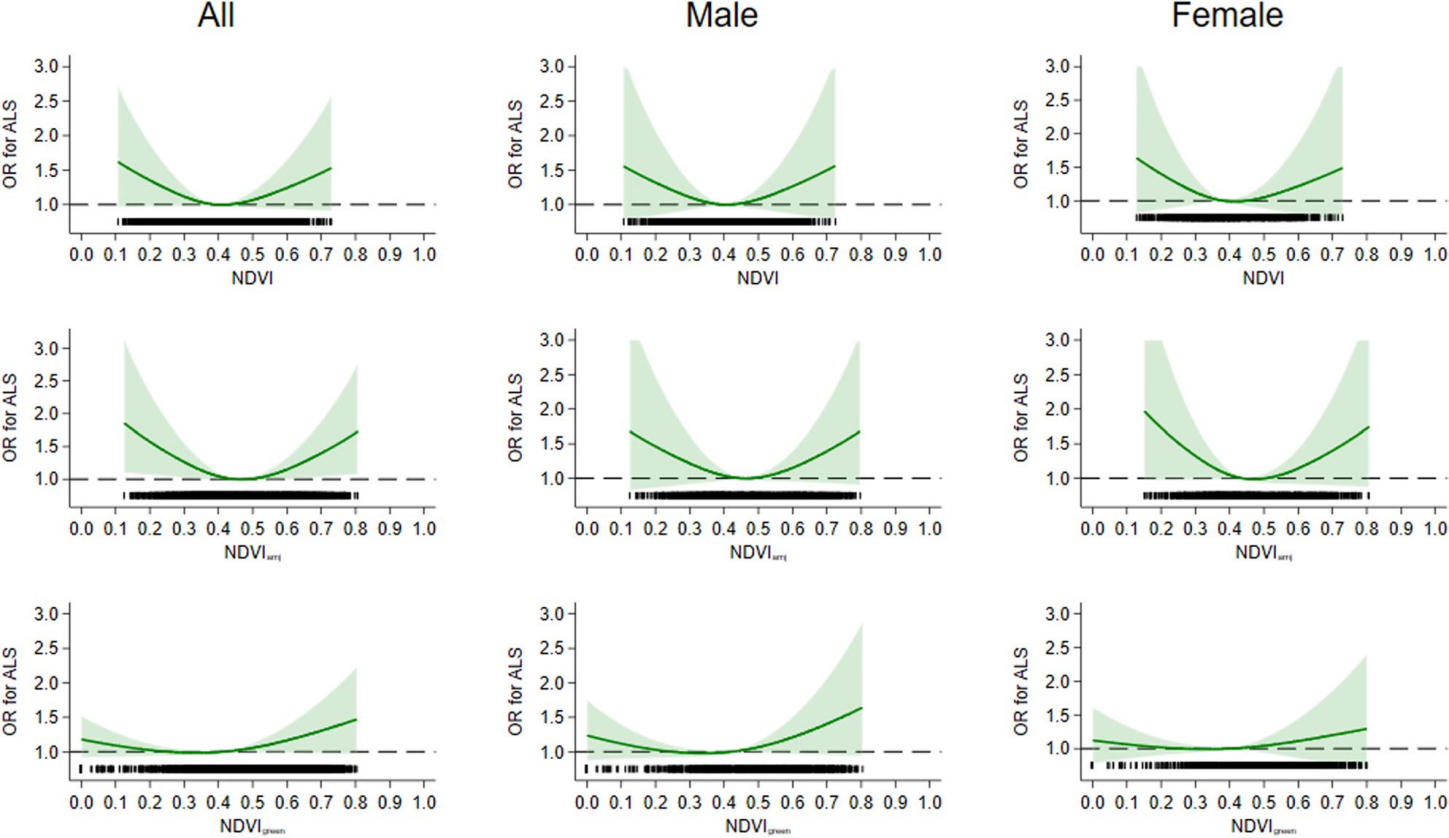
Amyotrophic lateral sclerosis (ALS) risk using odds ratio (OR) and correspondent 95% CIs, stratified by sex, for annual NDVI with 100 m radius buffer, spring seasonal NDVI_amj_ with 100 m radius buffer and spring seasonal NDVI_green_ with green buffer, calculated for each receptor considering a mobile mean from the year of diagnosis to the previous five years (line and area), and distribution of the values of annual NDVI with 100 m radius buffer, spring seasonal NDVI_amj_ with 100 m radius buffer and spring seasonal NDVI_green_ with green buffer of the mobile mean values for each subjects (pipe lines)

**Fig. 4:**
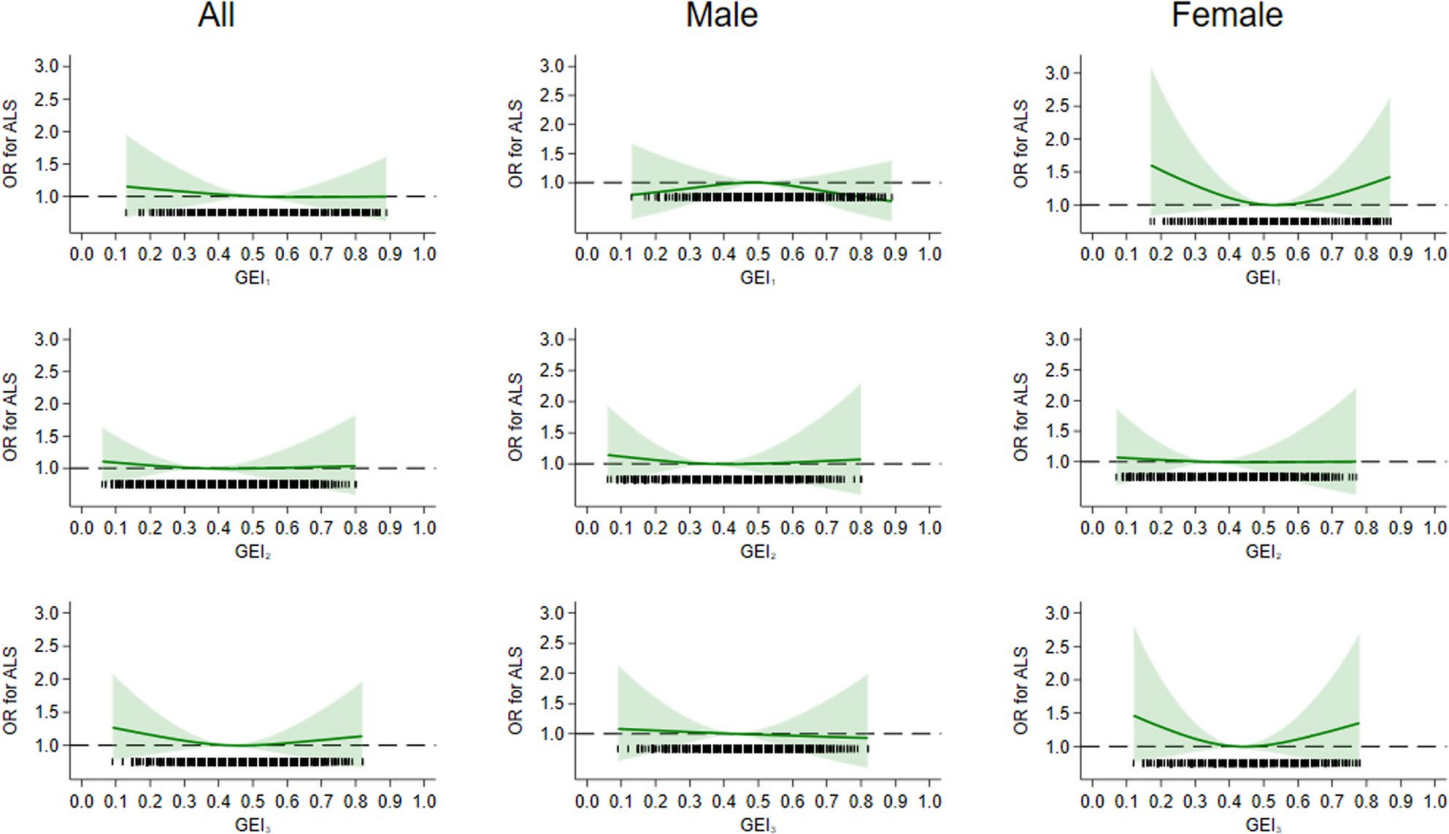
Amyotrophic lateral sclerosis (ALS) risk using odds ratio (OR) and correspondent 95% CIs, stratified by sex, for GEI_1_ with weighting factors $\:{w}_{1}=0.3,\:{w}_{2}=0.7$, GEI_2_ with $\:{w}_{1}=0.7,\:{w}_{2}=0.3$ and GEI_3_ with $\:{w}_{1}=0.5,\:{w}_{2}=0.5$, calculated for each receptor considering a mobile mean from the year of diagnosis to the previous five years (line and area), and distribution of the values of GEI_1_ ($\:{w}_{1}=0.3,\:{w}_{2}=0.7$), GEI_2_ ( $\:{w}_{1}=0.7,\:{w}_{2}=0.3$) and GEI_3_ ( $\:{w}_{1}=0.5,\:{w}_{2}=0.5$) of the mobile mean values for each subjects (pipe lines)

Figures 3 and 4 show the ALS risk curves according to NDVI and GEI indices. For NDVI, a U-shaped non-linear trend emerged, with increased ALS risk at both lower (< 0.2) and higher (> 0.6) NDVI levels, a pattern confirmed in sex-specific analysis. For GEI, mildly U-shaped curves emerged. For males, a similar result to the whole population was observable, with a different behavior just in the GEI_1_ case, due to the greater weight of accessibility. For females, a more defined U-shaped pattern was evident for both GEI_1_ and GEI_3_ with lower risk in the mid of the study range and higher values at the edges. ORs with their 95% CIs, at specific representative increments, are provided in the Supplementary Tables S2 and S3 for NDVI and GEI, respectively. Further adjusting for magnetic fields, LAN, and both factors yielded patterns substantially comparable to the unadjusted estimates for both NDVI and GEI (Supplementary Figures S2-S7). When adjusting for LAN, and consequently for both factors, a slight attenuation of NDVI, NDVI_amj_ and NDVI_green_ was observed for females, whereas a minor increase occurred in males (Supplementary Figures S3 and S4).

## Discussion

In this population-based case-control study conducted in Northern Italy, we evaluated the association between residential greenness exposure and the risk of ALS using multiple satellite-based and land-use derived indices. We observed a non-linear association between greenness and ALS risk, characterized by a U-shaped pattern. Specifically, ALS risk was higher at both low and high levels of greenness exposure, with lower risk at intermediate exposure levels.

Low greenness levels likely reflect urban environments characterized by higher pollution and psychosocial stress, whereas high levels may capture rural or agricultural settings potentially associated with increased exposure to pesticides or other environmental neurotoxins [7]. Several studies have documented an increased risk of ALS in urban areas characterized by higher levels of air pollution [21, 22, 46–53], and exposure to urban air pollution has also been associated with faster disease progression in ALS [54]. The role of air pollution as a possible mediator and/or confounder in ALS etiology, must therefore be considered due to its interplay with greenness [55, 56]. As for the other side of the U-shaped distribution, previous works and multiple studies spanning several decades have reported ALS clusters and higher incidence rates in rural settings, such as farming communities [57–68].

Specifically, for NDVI indices the increased ALS risk characterized both the lowest (< 0.2) and the highest (> 0.6) levels of exposure, with intermediate green space exposure appearing to be protective. Most epidemiological studies investigating greenness have reported beneficial associations with overall mortality and with neurodegenerative outcomes, including Parkinson’s disease or dementia, often attributing these effects to reduced air pollution, promotion of physical activity, psychological stress reduction, and enhanced social engagement [26–28, 31, 69–73]. The greater ALS risk associated with higher greenness exposure is not easily interpretable. A possible interpretation of this finding can be related to an increased exposure to pesticides or environmental chemicals used in agricultural settings [7, 36, 74]. Even though plausible, this hypothesis remains still speculative. The U-shaped association we observed could therefore be the result of an interplay of beneficial and detrimental mechanisms associated to greenness, as already disclosed for another neurodegenerative disease, dementia [75].

When using the GEI index, a U-shaped association could still be observed, even though more attenuated, due to the interplay of additional indices, such as green space accessibility. Of particular interest is the sex stratification of the risk for GEI_1_ and GEI_3_. The more pronounced U-shaped association observed among females may reflect sex-specific exposure patterns, differential residential exposure misclassification, or biological susceptibility, although these findings should be considered exploratory [76]. Alternatively, differential exposure misclassification by sex cannot be excluded, as residential greenness may reflect more accurately true environmental exposure for females than for males, likely having higher occupational mobility. Further studies should include not only residential exposure but a combination of it and job-place related factors.

Among the different NDVI versions analyzed, we designated NDVI_green_ as the reference index to be used for the GEI calculation. Our choice was also confirmed by the results obtained through interpolation maps, for which NDVI_green_ visually provides the most accurate distinction between urban and peripheral areas. On the contrary, NDVI yields the less defined distribution, probably due to its annual nature that flattens the vegetation signal quality. For GEI the same distinction capability can be well outlined in the scenario that favors vegetation (GEI_2_) and, even though more subtle, in the equal weighted one (GEI_3_). In GEI_1_ case instead, being accessibility the predominant contribution, a more homogeneous distribution was appreciated. This highlights a presence of accessible green areas well distributed in the municipalities under study, with a few exceptions in the more central urban areas [77].

This study presents some limitations. Residual confounding due to environmental and socioeconomic factors cannot be entirely excluded, although the matching by province may partially mitigate such risk. However, we adjusted for magnetic fields and LAN exposure, which are known as possible confounding factors [37, 44]. Moreover, greenness exposure was assessed at residential locations and may not fully capture individual-level exposure. Indeed, possible exposure misclassification may derive especially in relation to occupational or leisure activities. Residential history was not used to assess long term green space exposure, though residential mobility in Italy is lower than in other countries [78].

A strength of this study is the use of non-linear risk modelling across the entire range of green space exposure. This, allowed us to explore the full exposure–response relation and revealed non-linear associations that would have been obscured by linear modeling approaches, particularly when investigating environmental exposures with opposite dose-dependent health effects. Additional strengths of this study are the large number of cases, given disease rarity, and the population-based design without direct involvement of participants, thus avoiding selection bias. Furthermore, we used multiple greenness indices, both based on satellite data and land cover datasets, thus substantially strengthening exposure assessment.

## Conclusions

In conclusion, this study provides novel evidence of a non-linear association between residential greenness exposure and ALS risk, with increased risk at both low and high exposure levels. These findings highlight the need to consider the environmental context underlying green spaces, particularly in relation to urban, agricultural and rural exposures. Future studies integrating detailed data on pesticide use, air pollution, land-use practices, and individual mobility patterns will be essential to better define the relation between greenness and ALS, and to clarify the mechanisms underlying the observed associations.

## Supplementary Information

Supplementary Material 1.

## Data Availability

The data that have been used are confidential and are not publicly available due to restrictions on their containing information that could compromise the privacy of research participants.

## Abbreviations

A_i_: Accessibility
ALS: amyotrophic lateral sclerosis
GCR: Green Coverage Ratio
GEI: Green Exposure Index
LAN: Light at Night
NDVI: Normalized Difference Vegetation Index
