# Supplementary material for: Green space exposure and risk of amyotrophic lateral sclerosis: a population-based case-control study in Northern Italy
Source: Environ Health. 2026 May 22;25:61. doi: 10.1186/s12940-026-01311-w (PMC13371270; doi:10.1186/s12940-026-01311-w)
Supplement: Supplementary file 1 — Supplementary Material 1. [file 12940_2026_1311_MOESM1_ESM.docx]

**Supplementary Material**

**Supplementary Methods**

*Greenness indices*

The GCR index is land cover based and allows to quantify the amount of vegetation within an area of interest [1, 2]. This index, ranging from 0 to 1 in its standardized form, is computed as:

$$\begin{aligned} GCR=\left( \frac{A_{green}}{A_{tot}} \right)\#\left( 1 \right) \end{aligned}$$

where $A_{green}$ is the area filled with vegetation within the total area of study $A_{tot}$. The GCR index provides the ratio of vegetation within a defined area. Land cover maps provided by the Corine Land Cover program, elaborated and provided by the Emilia-Romagna Region geoportal, were employed to calculate the area ratio.

To estimate accessibility to green spaces, we developed an appositely tailored index, A_i_, with the expertise of our Department of Engineering. The A_i_ considers not only the availability of green spaces but also the distance of the receptor from the surrounding green areas and the quality of such areas [3-7].

In particular, a 15 minutes walking distance threshold was considered, ending in an approximate distance of 1200 meters by assuming an average walking speed of 4.8 km/h [8]. As for the quality of the green areas, the soil use and coverage provided by the Corine Land Cover maps was used to group out green areas into three main categories: artificial non-agricultural vegetated areas (e.g., urban parks), agricultural territories and forested and semi-natural environments. To each of these, accordingly to their correspondent public accessibility, an accessibility score P was assigned as described in the methodological article by Martini et al. [9].

The A_i_ index is obtained through the following function, with the accessibility decreasing with distance logarithmically from P to zero, (corresponding to the 15 minutes walkability limit):

$$\begin{aligned} A_{i}(d)=P\left[ 1-\frac{\ln\left( d \right)-\ln\left( 100 \right)}{\ln\left( 1300 \right)-\ln\left( 100 \right)} \right]\#\left( 2 \right) \end{aligned}$$

Discrete values of A_i_ were computed at intervals of 100 m from centroids of each green area, with all the points falling within the interval taking that value.

NDVI is a satellite-based index that expresses quantitative measures of vegetation density and health [10-12]. NDVI is obtained from reflectance values in the red and near-infrared spectral bands, exploiting plants absorption of visible light (from 0.4 to 0.7 µm), for use in photosynthesis, and reflectance in the near-infrared (0.7–1.1 μm). It can be computed as:

$$\begin{aligned} NDVI=\frac{NIR-RED}{NIR+RED} \#\left( 3 \right) \end{aligned}$$

Where NIR is the light reflected in the near-infrared and RED is the absorbed light in the red. The NDVI index displays values from -1 to +1, where positive values indicate presence of vegetation, zero corresponds to no vegetation, and negative values indicate the presence of water bodies. NDVI values lower than 0.1 are typical of sparsely or non-vegetated areas, such as barren areas of rock, sand, or snow. Intermediate values, between 0.2 and 0.5, represent moderately green areas, including shrub and grassland, whereas higher values, above 0.6, are characteristic of a healthy and dense vegetation, like temperate and tropical rainforests [13].

Finally, the GEI index, designed as a comprehensive index integrating all the above-mentioned ones, was defined as:

$$\begin{aligned} GEI=w_{1}\sqrt{NDVI\cdot GCR}+w_{2}A_{i}\#\left( 4 \right) \end{aligned}$$

where $w_{1}$ and $w_{2}$ are weighting factors determining the weight of each component in the computation of the whole GEI, such that their sum is always 1 [9]. Two main contributions were outlined: one related to vegetation, combining NDVI and GCR, and so vegetation density, quality and spatial extent, whereas the second to accessibility. Three different combinations of the weighting factors have been considered to test the sensitivity of GEI index to changing weights and to appreciate any underlying trend: the first one highlights the accessibility, with $w_{1}=0.3, w_{2}=0.7$ (GEI_1_); the second scenario empathizes the vegetation contribution to GEI with $w_{1}=0.7, w_{2}=0.3$ (GEI_2_); the last one provides the same weight to the two contribution $w_{1}=0.5, w_{2}=0.5$ (GEI_3_). The GEI index is dimensionless and assumes values going from 0 to 1.

*Calculation procedure*

For each receptor the GCR index was calculated for each year under study using Eq. 1 as described above.

The accessibility A_i_ was calculated through a custom workflow implemented in QGIS [14]. From the centroid of each green polygon a 1300 meters buffer was created and divided at intervals of 100 m in 13 rings. Each ring was assigned with the correspondent value of A_i_, computed through Eq. 2, considering the interval edge value as the distance. Within the first ring therefore, the maximum value of A_i_ is accomplished, with $A_{i}=P$, while in the last one (13th) the lowest value, corresponding to zero, is reached. Intersecting the accessibility buffers with the receptor geocoded locations, each receptor was assigned with the accessibility value of the ring it fell into, or, in those cases in which it fell into more rings, with the maximum value among them.

NDVI was calculated from the images provided by the Landsat series satellites. In particular, the images acquired by Landsat 5, Landsat 7 and Landsat 8 were used, to cover a total of 18 years, from 1993 to 2011.

A Google Earth Engine procedure was developed to extract the NDVI values ​​associated with each receptor. The satellite images have been reprocessed to remove unwanted conditions such as clouds, cloud shadows and cirrus clouds, and afterward the indices have been calculated for each pixel of each image [9]. The annual mean of the values obtained was calculated to have a single reference image for each year.

To better represent the vegetation condition, being vegetation indices extremely sensitive to seasonal variations, also a seasonal metric was computed to be compared with the annual one. Spring season, displaying the vegetation peak, seemed the most indicated one to be used and images from April, May and June (amj) were exploited for the NDVI computation as described above. The spring seasonal index will be referred to as NDVI_amj_.

The exposure for each receptor was obtained by combining the geographic coordinates with the processed satellite images, and was determined within a buffer centered in each receptor residence [15, 16]. Among the eligible buffer sizes that can be found in literature, we chose to employ a circular buffer of radius 100 m, as we also did in previous studies [17, 18]. Our choice was also due to computational reasons and was supported by literature since no significant difference in exposure evaluation was observed with a 200 m radius [9].

Additionally, an alternative buffer was considered for the seasonal approach, the so called “green buffer”. The green buffer is the product of the interpolation of a circular buffer (100 m radius) and of the polygons classified as green spaces in the Corine Land Cover maps for land use. Consequently, unlike conventional circular buffers, the green one includes only those areas that are classified as green ones and allows to calculate NDVI only upon them, providing a more refined spatial aggregation. This NDVI calculated with the green buffer will be called NDVI_green_.

Despite the increased refinement in the exposure evaluation accessed with the seasonal restricted analysis and the use of green buffers, some drawbacks arise from this approach. The satellite exploited to acquire the necessary data cover the area of study at regular time intervals of 16 days, and cloud filters are applied to them (i.e., if the area is completely covered by clouds, no data can be acquired). Furthermore, having reduced the annual time window a reduced amount of satellite data was available and consequently the eventual presence of data holes became relevant. In particular, with this approach, some of the years of interest for the study (1991, 1994 and 1999) were completely holed. To deal with the presence of these holed years and with the single data holes present in other years, a procedure to fill them was developed. In the case of an isolated data hole, the mean of the previous year and next year values was calculated and substituted. For those receptors displaying adjacent holes for consecutive years, the mean among the first available previous year and of the next available year was used. This method however was applied only if the gap extended at most for 5 consecutive data holes, assuming that no significant variations of green spaces could occur in a sufficiently restrained interval of time. The holes that could not be filled through this method were considered as missing points. The same issue, but less outstanding was observed even with the sole seasonal restricted analysis. Still the filling data holes procedure was applied even to NDVI_amj_.

At the end of the computation procedure an average value of NDVI for each buffer and for each year taken into account, for all three different NDVI index versions described above (NDVI, NDVI_amj_, NDVI_green_), was obtained, representing the exposure to the green areas of each case study for each year.

For the GEI calculation the NDVI_green_ index was used, having been identified as the most suitable vegetation proxy among the ones tested in this study [9]. For each receptor and for each year of study the GEI index was calculated with the three weighting factor combinations described above using Eq. 4. Being NDVI_green_ affected by the presence of not fillable data holes, even GEI index, in all three combinations displayed them.

**Supplementary Tables**

**Table S1.** Characteristics of study participants, stratified by sex and category, for age at diagnosis and province of residence; median and interquartile range (IQR) for cases and controls stratified by sex for GCR, A_i_, annual NDVI with 100 m radius buffer, spring seasonal NDVI_amj_ with 100 m radius buffer and spring seasonal NDVI_green_ with green buffer GEI_1_ with weighting factors $w_{1}=0.3, w_{2}=0.7$, GEI_2_ with $w_{1}=0.7, w_{2}=0.3$ and GEI_3_ with $w_{1}=0.5, w_{2}=0.5$.

|  | **Males** | | **Females** | |
| --- | --- | --- | --- | --- |
|  | **Cases** | **Controls** | **Cases** | **Controls** |
|  | **N (%)** | **N (%)** | **N (%)** | **N (%)** |
| **Total** | 269 (100) | 1038 (100) | 897 (100) | 230 (100) |
| **Age at diagnosis (years)** |  |  |  |  |
| Median (IQR) | 66 (59 - 74) | 66 (59 - 74) | 71.5 (63 - 78) | 72 (64 - 78) |
| <55 | 45 (16.73) | 170 (16.38) | 29 (12.61) | 111 (12.37) |
| 55-74 | 164 (60.97) | 636 (61.27) | 109 (47.39) | 427 (47.60) |
| ≥74 | 60 (22.30) | 232 (22.35) | 92 (40.00) | 359 (40.02) |
| **Province** |  |  |  |  |
| Modena | 126 (46.84) | 480 (46.24) | 109 (47.39) | 425 (47.38) |
| Reggio Emilia | 82 (30.48) | 322 (31.02) | 70 (30.43) | 277 (30.88) |
| Parma | 61 (22.68) | 236 (22.74) | 51 (22.17) | 195 (21.74) |
|  | **Median (IQR)** | **Median (IQR)** | **Median (IQR)** | **Median (IQR)** |
| **GCR** | 0.111 (0 - 0.336) | 0.110 (0 - 0.365) | 0.097 (0 - 0.299) | 0.085 (0 - 0.299) |
| **A_i_** | 0.572 (0.460 - 0.730) | 0.572 (0.460 - 0.730) | 0.572 (0.460 - 0.730) | 0.572 (0.460 - 0.730) |
| **NDVI** |  |  |  |  |
| NDVI | 0.400 (0.315 - 0.507) | 0.398 (0.318 - 0.503) | 0.394 (0.311 - 0.482) | 0.383 (0.312 - 0.487) |
| NDVI_amj_ | 0.451 (0.366 - 0.594) | 0.456 (0.361 - 0.580) | 0.448 (0.357 - 0.558) | 0.436 (0.362 - 0.560) |
| NDVI_green_ | 0.414 (0 - 0.618) | 0.423 (0 - 0.580) | 0.419 (0 - 0.567) | 0.396 (0 - 0.564) |
| **GEI** |  |  |  |  |
| GEI_1_ | 0.499 (0.400 - 0.579) | 0.499 (0.400 - 0.581) | 0.498 (0.400 - 0.583) | 0.511 (0.400 - 0.585) |
| GEI_2_ | 0.358 (0.172 - 0.489) | 0.362 (0.181 - 0.504) | 0.345 (0.172 - 0.487) | 0.338 (0.187 - 0.491) |
| GEI_3_ | 0.430 (0.286 - 0.538) | 0.431 (0.286 - 0.535) | 0.410 (0.286 - 0.549) | 0.424 (0.286 - 0.540) |

**Table S2.** Odds ratios (ORs) with their 95% confidence intervals (CIs) NDVI, NDVI_amj_ and NDVI_green_ at specific representative increments: 0.20, 0.40 and 0.60. The choice of these values was driven by the visual threshold values below (0.2) and above (0.6) which an increment of the indices could be observed; moreover, we decided to take an additional descriptive point in between such interval, which corresponds to the 0.40 value.

|  | **OR (95% CI)** | **OR (95% CI)** | **OR (95% CI)** |
| --- | --- | --- | --- |
| **All** | | | |
| **NDVI** | **0.20** | **0.40** | **0.60** |
| NDVI | 1.35 (0.97-1.87) | 1.00 (0.99-1.00) | 1.24 (0.93-1.65) |
| NDVI_amj_ | 1.57 (1.07-2.31) | 1.05 (1.00-1.10) | 1.15 (0.99-1.33) |
| NDVI_green_ | 1.02 (0.94-1.12) | 1.00 (0.99-1.00) | 1.17 (0.98-1.38) |
| **Males** | | | |
| **NDVI** | **0.20** | **0.40** | **0.60** |
| NDVI | 1.31 (0.84-2.05) | 1.00 (1.00-1.00) | 1.26 (0.86-1.85) |
| NDVI_amj_ | 1.46 (0.86-2.46) | 1.04 (0.97-1.12) | 1.15 (0.95-1.39) |
| NDVI_green_ | 1.03 (0.91-1.15) | 0.99 (0.97-1.00) | 1.21 (0.97-1.51) |
| **Females** | | | |
| NDVI | **0.20** | **0.40** | **0.60** |
| NDVI | 1.40 (0.87-2.25) | 0.99 (0.97-1.01) | 1.22 (0.80-1.87) |
| NDVI_amj_ | 1.72 (0.98-3.02) | 1.05 (0.99-1.11) | 1.14 (0.92-1.43) |
| NDVI_green_ | 1.02 (0.90-1.15) | 1.00 (1.00-1.00) | 1.11 (0.86-1.45) |

**Table S3.** Odds ratios (ORs) with their 95% confidence intervals (CIs) GEI_1_, GEI_2_ and GEI_3_ at specific representative increments: 0.25, 0.50 and 0.75. The choice of these values was driven by the visual threshold values below (0.25) and above (0.75) which an increment of the indices could be observed; moreover, we decided to take an additional descriptive point in between interval, which corresponds to the 0.50 value.

|  | **OR (95% CI)** | **OR (95% CI)** | **OR (95% CI)** |
| --- | --- | --- | --- |
| **All** | | | |
| **GEI** | **0.25** | **0.50** | **0.75** |
| GEI_1_ | 1.10 (0.78-1.54) | 1.00 (1.00-1.01) | 0.99 (0.75-1.31) |
| GEI_2_ | 1.03 (0.94-1.13) | 1.00 (0.88-1.13) | 1.03 (0.63-1.68) |
| GEI_3_ | 1.11 (0.89-1.39) | 1.00 (0.95-1.06) | 1.10 (0.71-1.71) |
| **Males** | | | |
| **GEI** | **0.25** | **0.50** | **0.75** |
| GEI_1_ | 0.87 (0.54-1.39) | 1.00 (1.00-1.00) | 0.80 (0.53-1.21) |
| GEI_2_ | 1.04 (0.91-1.18) | 1.00 (0.86-1.18) | 1.06 (0.55-2.05) |
| GEI_3_ | 1.04 (0.77-1.41) | 0.99 (0.91-1.07) | 0.94 (0.51-1.73) |
| **Females** | | | |
| GEI | **0.25** | **0.50** | **0.75** |
| GEI_1_ | 1.41 (0.87-2.28) | 1.00 (0.99-1.01) | 1.22 (0.84-1.76) |
| GEI_2_ | 1.02 (0.90-1.16) | 0.99 (0.81-1.21) | 1.00 (0.48-2.11) |
| GEI_3_ | 1.20 (0.87-1.65) | 1.01 (0.93-1.11) | 1.31 (0.70-2.43) |

**Supplementary Figures**

**Figure S1.** Municipal boundaries (yellow solid line) and study area boundaries (red solid line) of the three Provinces investigated: a) Parma; b) Reggio Emilia; c) Modena.

**
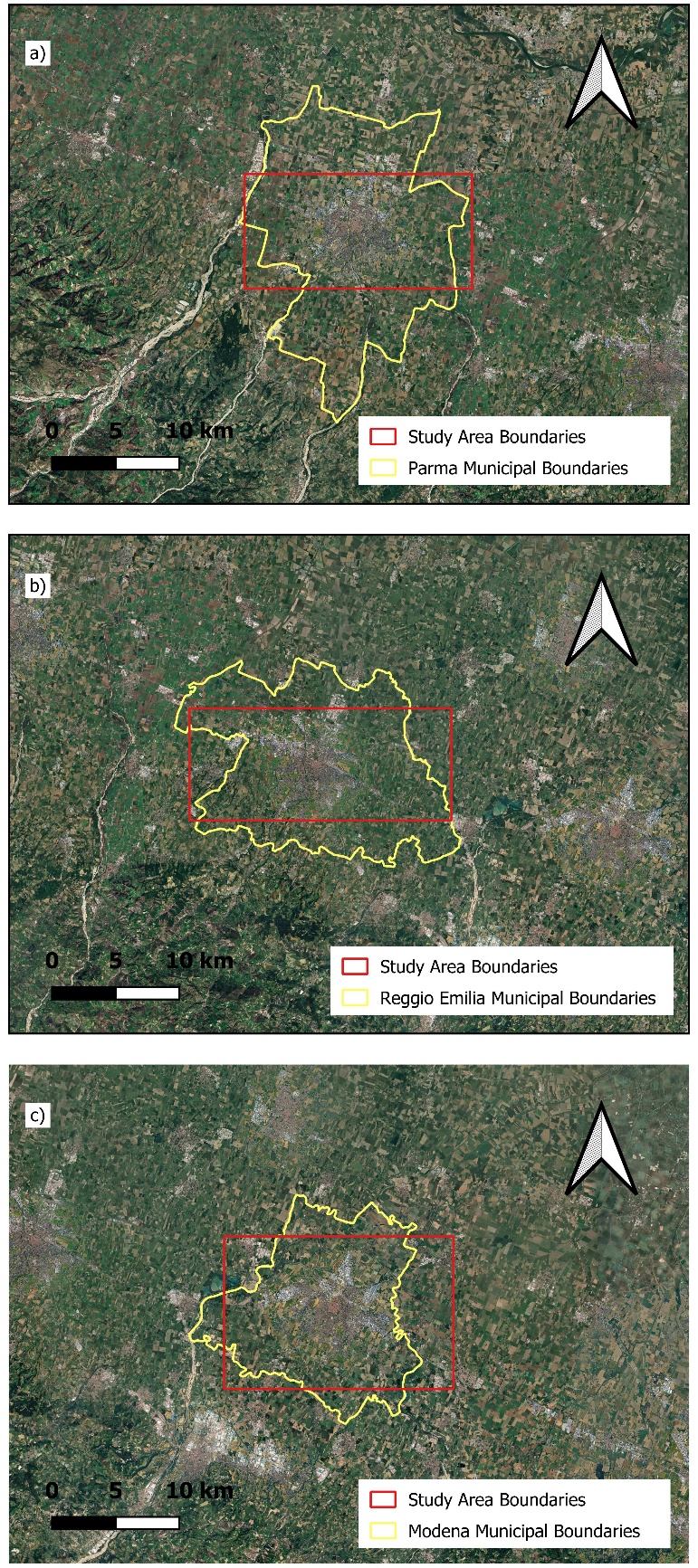
**

**Figure S2.** Amyotrophic lateral sclerosis (ALS) risk using odds ratio (OR) and correspondent 95% CIs, stratified by sex and adjusted for Magnetic Fields, for annual NDVI with 100 m radius buffer, spring seasonal NDVI_amj_ with 100 m radius buffer and spring seasonal NDVI_green_ with green buffer, calculated for each receptor considering a mobile mean from the year of diagnosis to the previous five years (line and area), and distribution of the values of annual NDVI with 100 m radius buffer, spring seasonal NDVI_amj_ with 100 m radius buffer and spring seasonal NDVI_green_ with green buffer of the mobile mean values for each subjects (pipe lines).


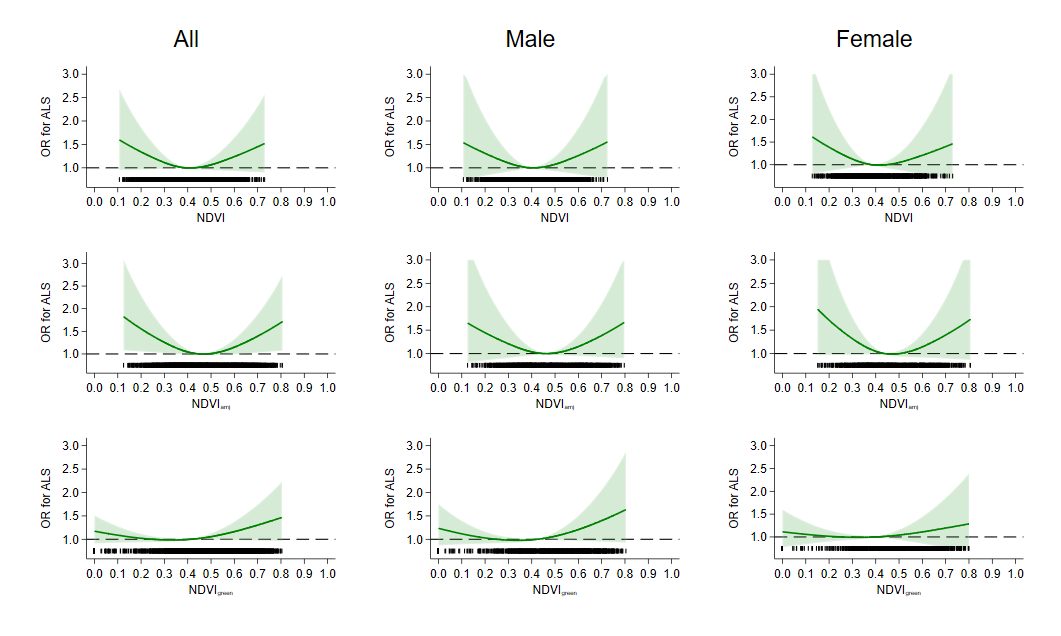


**Figure S3.** Amyotrophic lateral sclerosis (ALS) risk using odds ratio (OR) and correspondent 95% CIs, stratified by sex and adjusted for LAN, for annual NDVI with 100 m radius buffer, spring seasonal NDVI_amj_ with 100 m radius buffer and spring seasonal NDVI_green_ with green buffer, calculated for each receptor considering a mobile mean from the year of diagnosis to the previous five years (line and area), and distribution of the values of annual NDVI with 100 m radius buffer, spring seasonal NDVI_amj_ with 100 m radius buffer and spring seasonal NDVI_green_ with green buffer of the mobile mean values for each subjects (pipe lines).


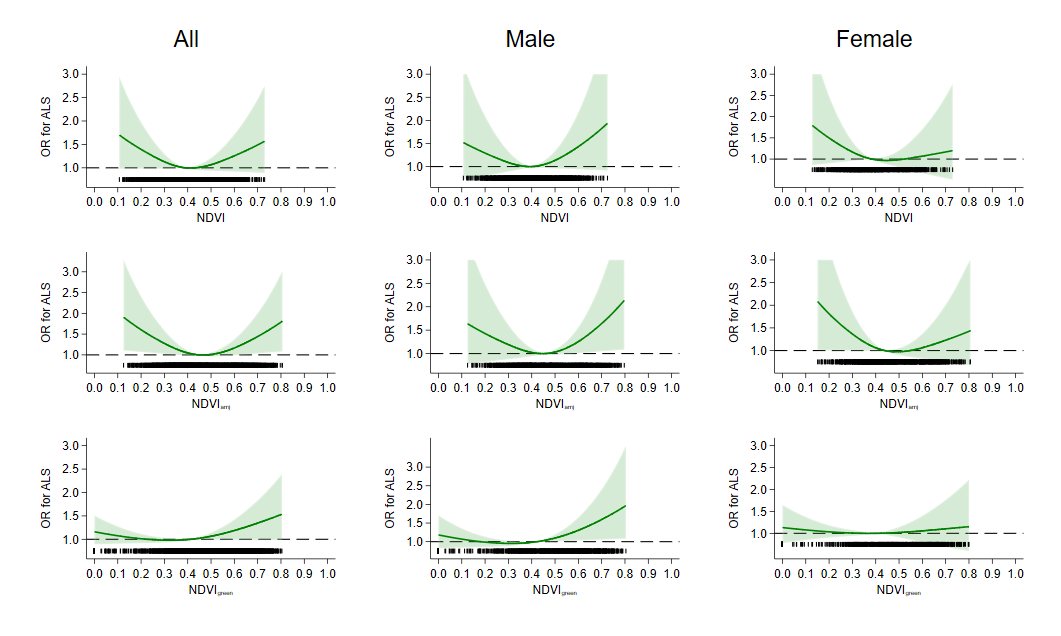


**Figure S4.** Amyotrophic lateral sclerosis (ALS) risk using odds ratio (OR) and correspondent 95% CIs, stratified by sex and adjusted for Magnetic Fields and LAN, for annual NDVI with 100 m radius buffer, spring seasonal NDVI_amj_ with 100 m radius buffer and spring seasonal NDVI_green_ with green buffer, calculated for each receptor considering a mobile mean from the year of diagnosis to the previous five years (line and area), and distribution of the values of annual NDVI with 100 m radius buffer, spring seasonal NDVI_amj_ with 100 m radius buffer and spring seasonal NDVI_green_ with green buffer of the mobile mean values for each subjects (pipe lines).


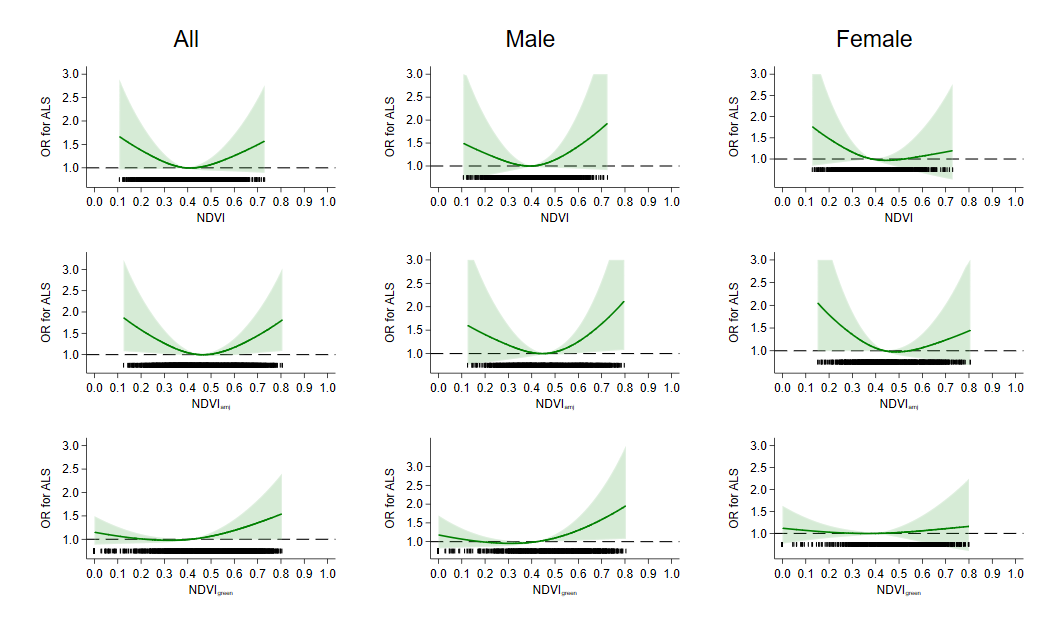


**Figure S5.** Amyotrophic lateral sclerosis (ALS) risk using odds ratio (OR) and correspondent 95% CIs, stratified by sex and adjusted for Magnetic Fields, for GEI_1_ with weighting factors $w_{1}=0.3, w_{2}=0.7$, GEI_2_ with $w_{1}=0.7, w_{2}=0.3$ and GEI_3_ with $w_{1}=0.5, w_{2}=0.5$, calculated for each receptor considering a mobile mean from the year of diagnosis to the previous five years (line and area), and distribution of the values of GEI_1_ ($w_{1}=0.3, w_{2}=0.7$), GEI_2_ ( $w_{1}=0.7, w_{2}=0.3$) and GEI_3_ ( $w_{1}=0.5, w_{2}=0.5$) of the mobile mean values for each subjects (pipe lines).


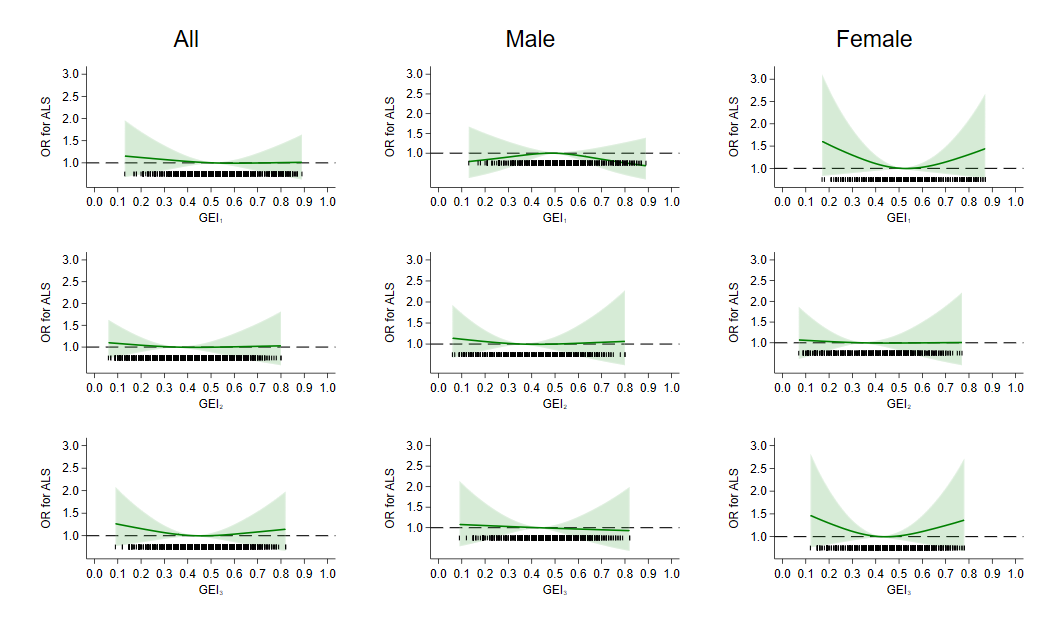


**Figure S6.** Amyotrophic lateral sclerosis (ALS) risk using odds ratio (OR) and correspondent 95% CIs, stratified by sex and adjusted for LAN, for GEI_1_ with weighting factors $w_{1}=0.3, w_{2}=0.7$, GEI_2_ with $w_{1}=0.7, w_{2}=0.3$ and GEI_3_ with $w_{1}=0.5, w_{2}=0.5$, calculated for each receptor considering a mobile mean from the year of diagnosis to the previous five years (line and area), and distribution of the values of GEI_1_ ($w_{1}=0.3, w_{2}=0.7$), GEI_2_ ( $w_{1}=0.7, w_{2}=0.3$) and GEI_3_ ( $w_{1}=0.5, w_{2}=0.5$) of the mobile mean values for each subjects (pipe lines).


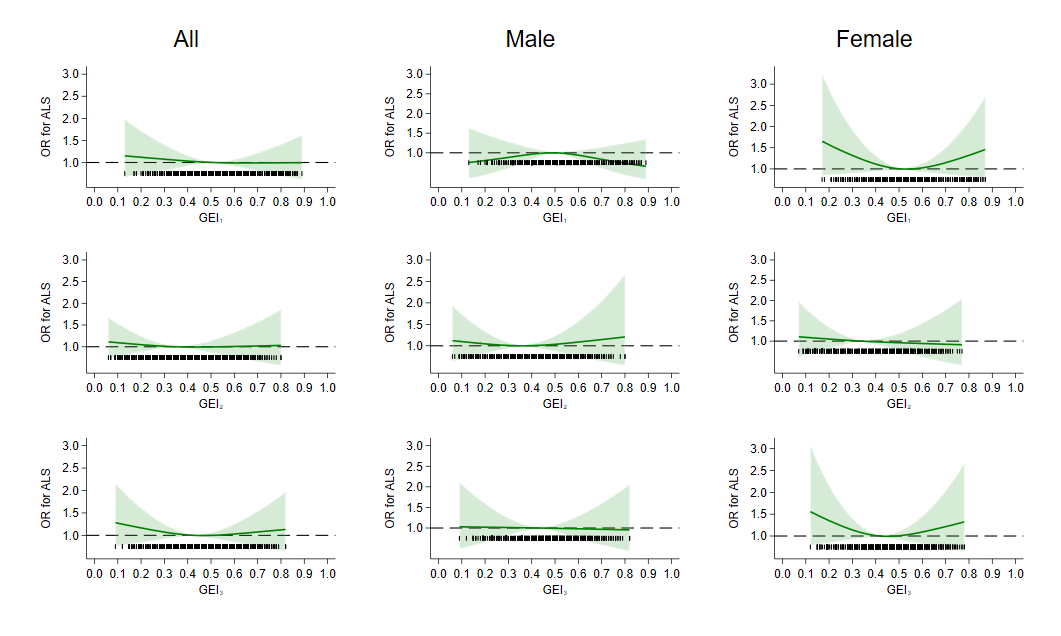


**Figure S7.** Amyotrophic lateral sclerosis (ALS) risk using odds ratio (OR) and correspondent 95% CIs, stratified by sex and adjusted for Magnetic Fields and LAN, for GEI_1_ with weighting factors $w_{1}=0.3, w_{2}=0.7$, GEI_2_ with $w_{1}=0.7, w_{2}=0.3$ and GEI_3_ with $w_{1}=0.5, w_{2}=0.5$, calculated for each receptor considering a mobile mean from the year of diagnosis to the previous five years (line and area), and distribution of the values of GEI_1_ ($w_{1}=0.3, w_{2}=0.7$), GEI_2_ ( $w_{1}=0.7, w_{2}=0.3$) and GEI_3_ ( $w_{1}=0.5, w_{2}=0.5$) of the mobile mean values for each subjects (pipe lines).


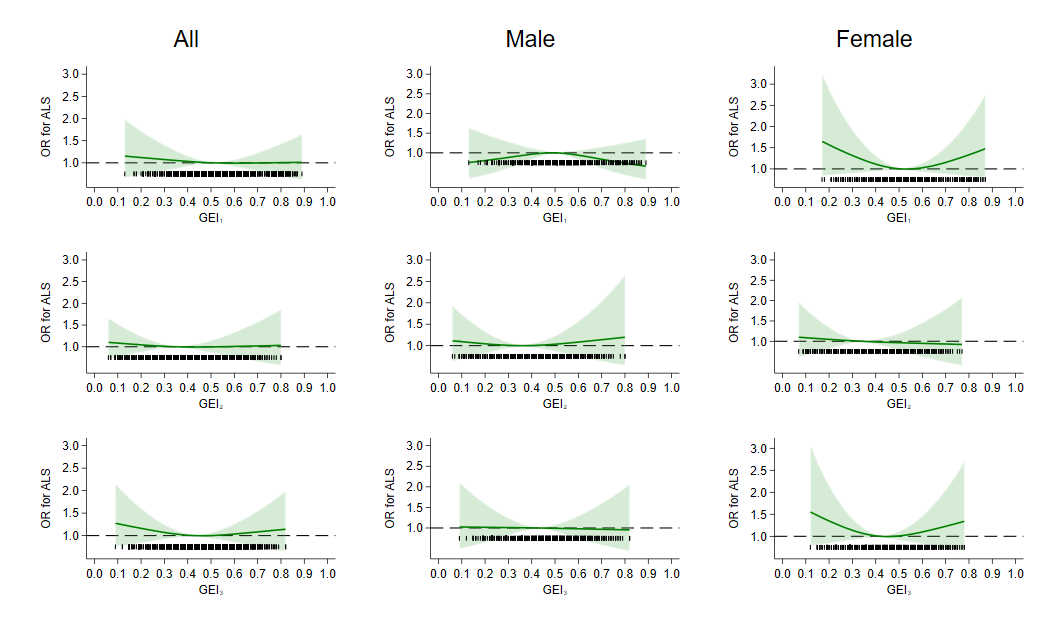


**References**

1. Cao Y, Li G, Huang Y: Spatiotemporal Evolution of Residential Exposure to Green Space in Beijing. Remote Sensing 2023, 15(6):1549. <https://doi.org/10.3390/rs15061549>.

2. Tian Y, Jim CY, Tao Y, Shi T: Landscape ecological assessment of green space fragmentation in Hong Kong. Urban For Urban Green 2011, 10(2):79-86. <https://doi.org/10.1016/j.ufug.2010.11.002>.

3. Moreno C, Allam Z, Chabaud D, Gall C, Pratlong F: Introducing the “15-Minute City”: Sustainability, Resilience and Place Identity in Future Post-Pandemic Cities. Smart Cities 2021, 4(1):93-111. <https://doi.org/10.3390/smartcities4010006>.

4. Barbosa O, Tratalos JA, Armsworth PR, Davies RG, Fuller RA, Johnson P, Gaston KJ: Who benefits from access to green space? A case study from Sheffield, UK. Landscape and Urban Planning 2007, 83(2-3):187-195. <https://doi.org/10.1016/j.landurbplan.2007.04.004>.

5. Villanueva-Durbán N, Lorenzo-Sáez E, Lerma-Arce V, Coll-Aliaga E: Evaluation of Urban Accessibility Through Geomarketing Techniques: Case Study in Valencia (Spain). ISPRS Int J Geo-Inf 2025, 14(2):60. <https://doi.org/10.3390/ijgi14020060>.

6. Kwan MP, Weber J: Individual Accessibility Revisited: Implications for Geographical Analysis in the Twenty-first Century. Geographical Analysis 2003b, 35(4):341-353. <https://doi.org/10.1111/j.1538-4632.2003.tb01119.x>.

7. Kwan MP, Weber J: Evaluating the Effects of Geographic Contexts on Individual Accessibility: A Multilevel Approach. Urban Geogr 2003a, 24(8):647–671. <https://doi.org/10.2747/0272-3638.24.8.647>.

8. Seyfried A, Steffen B, Klingsch W, Boltes M: The fundamental diagram of pedestrian movement revisited. J Stat Mech: Theory Exp 2005, 2005(10):P10002-P10002. <https://doi.org/10.1088/1742-5468/2005/10/p10002>.

9. Martini N, Despini F, Filippini T, Vinceti M, Teggi S, Costanzini S: Assessing green space exposure: from traditional metrics to the Green Exposure Index (GEI) with application to a Northern Italy residential dataset. Environ Res 2026, 298:124254. <https://doi.org/10.1016/j.envres.2026.124254>.

10. Huang S, Tang L, Hupy JP, Wang Y, Shao G: A commentary review on the use of normalized difference vegetation index (NDVI) in the era of popular remote sensing. Journal of Forestry Research 2020, 32(1):1-6. <https://doi.org/10.1007/s11676-020-01155-1>.

11. Pettorelli N, Vik JO, Mysterud A, Gaillard JM, Tucker CJ, Stenseth NC: Using the satellite-derived NDVI to assess ecological responses to environmental change. Trends Ecol Evol 2005, 20(9):503-510. <https://doi.org/10.1016/j.tree.2005.05.011>.

12. Xu Y, Yang Y, Chen X, Liu Y: Bibliometric Analysis of Global NDVI Research Trends from 1985 to 2021. Remote Sensing 2022, 14(16):3967. <https://doi.org/10.3390/rs14163967>.

13. Measuring Vegetation (NDVI & EVI) [<http://earthobservatory.nasa.gov/Features/MeasuringVegetation/measuring_vegetation_2.php>]

14. QGIS Geographic Information System. Open Source Geospatial Foundation. [<https://qgis.org/>]

15. Malagoli C, Costanzini S, Heck JE, Malavolti M, De Girolamo G, Oleari P, Palazzi G, Teggi S, Vinceti M: Passive exposure to agricultural pesticides and risk of childhood leukemia in an Italian community. Int J Hyg Environ Health 2016, 219(8):742-748. <https://doi.org/10.1016/j.ijheh.2016.09.015>.

16. Wittich KP, Siebers J: Aerial short-range dispersion of volatilized pesticides from an area source. Int J Biometeorol 2002, 46(3):126-135. <https://doi.org/10.1007/s00484-002-0125-3>.

17. Vinceti M, Filippini T, Violi F, Rothman KJ, Costanzini S, Malagoli C, Wise LA, Odone A, Signorelli C, Iacuzio L *et al*: Pesticide exposure assessed through agricultural crop proximity and risk of amyotrophic lateral sclerosis. Environ Health 2017, 16(1):91. <https://doi.org/10.1186/s12940-017-0297-2>.

18. Dadvand P, de Nazelle A, Figueras F, Basagana X, Su J, Amoly E, Jerrett M, Vrijheid M, Sunyer J, Nieuwenhuijsen MJ: Green space, health inequality and pregnancy. Environ Int 2012, 40:110-115. <https://doi.org/10.1016/j.envint.2011.07.004>.
